# Prediction of the potentially suitable areas of *Ligularia virgaurea* and *Ligularia sagitta* on the Qinghai–Tibet Plateau based on future climate change using the MaxEnt model

**DOI:** 10.3389/fpls.2023.1193690

**Published:** 2023-07-20

**Authors:** Rui Dong, Li-min Hua, Rui Hua, Guo-hui Ye, Darhan Bao, Xin-cheng Cai, Bin Cai, Xi-cun Zhao, Bin Chu, Zhuang-sheng Tang

**Affiliations:** Key Laboratory of Grassland Ecosystem of the Ministry of Education, College of Grassland Science, Gansu Agricultural University, Engineering and Technology Research Centre for Alpine Rodent Pest Control, National Forestry and Grassland Administration, Lanzhou, China

**Keywords:** *Ligularia virgaurea*, *Ligularia sagitta*, climate change, MaxEnt, suitable habitat, Qinghai-Tibet Plateau

## Abstract

*Ligularia virgaurea* and *Ligularia sagitta* are two species of poisonous plants with strong invasiveness in natural grasslands in China that have caused considerable harm to animal husbandry and the ecological environment. However, little is known about their suitable habitats and the key environmental factors affecting their distribution. Although some studies have reported the distributions of poisonous plants on the Qinghai–Tibet Plateau (QTP) and predicted their potential distributions at local scales in some regions under climate change, there have been few studies on the widespread distributions of *L. virgaurea* and *L. sagitta*. In this study, we recorded 276 and 118 occurrence points of *L. virgaurea* and *L. sagitta* on the QTP using GPS, and then used the MaxEnt model to predict the distribution of suitable habitats. Results showed that (1) under current climate conditions, *L. virgaurea* and *L. sagitta* are mainly distributed in southern Gansu, eastern Qinghai, northwestern Sichuan, eastern Tibet, and southwestern Yunnan, accounting for approximately 34.9% and 39.8% of the total area of the QTP, respectively; (2) the main environmental variables affecting the distribution of suitable habitats for *L. virgaurea* and *L. sagitta* are the Human Footprint Index (52.8%, 42.2%), elevation (11%, 4.4%), soil total nitrogen (18.9%, 4.2%), and precipitation seasonality (5.1%, 7.3%); and (3) in the future, in the 2050s and 2070s, the area of habitat of intermediate suitability for *L. virgaurea* will spread considerably in northwest Sichuan, while that of high suitability for *L. sagitta* will spread to eastern Tibet and western Sichuan.

## 1 Introduction

Climate is a major determinant of the range and distribution of plant species (Yang et al., 2022a). Climate change will lead to changes in the suitable ranges of some species and accelerate the decline in global biodiversity (Huang et al., 2021). Both native and non-native species have the ability to modify habitats and ecological processes, which not only poses serious threats to biodiversity and ecological processes, but also affects the composition and structure of plant communities (Jamil et al., 2022). After habitat degradation, invasive species are the second leading cause of biodiversity loss due to their ability to compete with and replace native plants (Haq et al., 2022). Human activity is one of the important ways that species invasion can be caused. The spread of invasive species to new ranges is reducing species diversity on Earth, which is already at unsustainable levels (Kariyawasam et al., 2019). Furthermore, in addition to biodiversity, species invasions can have negative impacts on agriculture, forestry, and human health (Hallgren et al., 2019). Long-term effects could pose a significant threat to global economic, social and environmental stability (Neven et al., 2018). In recent decades, the number of invasive species has continued to increase, especially in China’s natural grassland ecosystems (Shen et al., 2021; Shen et al., 2022; Yang et al., 2022b). Invasive species have become an important factor affecting the sustainable development of grassland animal husbandry (Wang et al., 2019). Therefore, a better understanding of the link between invasive species and climate change is crucial, especially for species that have a significant impact on global biodiversity.

In recent years, species distribution models (SDMs) have become an important tool for studying species distribution patterns. They combine known distribution points and corresponding environmental variables to simulate the geographic distribution of species and the response to climate change based on certain algorithms (Sun et al., 2020). Among those SDMs currently available, the MaxEnt model, developed by (Phillips et al., 2006) and based on the principle of maximum entropy, performs best in simulating the geographical distribution of species. The main advantage of such an approach is that a high degree of accuracy and stability can be maintained even in the case of a partial lack of species data or small sample size (Wang et al., 2021). In addition, MaxEnt also offers rapid calculation speeds and flexible operation (Phillips et al., 2006; Hu et al., 2020); plus, its predictions can be clearly and intuitively visualized through a geographic information system (Moreno et al., 2011). MaxEnt is currently the most widely used SDM, and many studies have demonstrated its reliability in predicting species distributions (Ancillotto et al., 2019; Ab Lah et al., 2021; Tang et al., 2021). In China, such research on the suitable distribution area of invasive species, such as *Sporobolus alterniflorus*, *Flaveria bidentis*, and *Ageratina adenophora*, provides an important reference in the control of biological invasions and the protection of biodiversity (Yuan et al., 2021; Liu et al., 2022b; Wei et al., 2022). In terms of natural grassland protection in China, research on the suitable distribution area of *Pedicularis kansuensis*, for example, has provided strong support for grassland managers to deal with the large-scale spread of poisonous plants in natural grasslands and reduce the risk of invasion (Wang et al., 2019).

China’s grassland area is the second largest in the world, and grassland is the largest type of terrestrial ecosystem in China, accounting for 41.7% of the total land area (Paoletti, 2005). The ecological, economic and social functions of grasslands play an important role in the development of human society (Egoh et al., 2011). The Qinghai–Tibet Plateau (QTP) has the largest area of grassland in China and plays an important role in grassland animal husbandry and the construction of ecological barriers (Wang et al., 2016; Li et al., 2019). Grassland animal husbandry is an important source of support for the livelihoods of local herdsmen. However, a survey found that the current natural grassland area of poisonous plants in China is approximately 4.504×10^7^m^2^, accounting for around 11.3% of the total natural grassland area (Zhao et al., 2008; Huang and Shang, 2019). This will seriously restrict the development of grassland animal husbandry, and thus even affect the stability of people’s lives in Tibetan areas. *Ligularia virgaurea* and *Ligularia sagitta* are perennial poisonous herbaceous plants in the family Asteraceae. They mainly grow in northeastern Tibet, northwestern Yunnan, Sichuan, Qinghai, and Gansu. These two plants are mainly cloned and propagated, and are endemic to China (Ma et al., 2006). Due to their own reproductive advantages, *L. virgaurea* and *L. sagitta* have strong adaptability to adverse environments, and the two plants contain poisonous substances, which lead to food refusal behavior in livestock (Xie et al., 2014; Ade et al., 2021). In addition, *L. virgaurea* and *L. sagitta* the growth of good-quality pastures through allelopathy, meaning more resources can be used for reproduction (Zhang et al., 2011). Therefore, they can exist in large areas and spread rapidly in grasslands. To date, there have been few studies on the potential suitable distribution of poisonous plants on the QTP. Thus, studying the distribution ranges and occurrence areas of these two species of *Ligularia* is of great significance for preventing local poisonous plants from invading healthy grasslands, and can help grassland managers reduce the risk of intrusion into grassland vegetation.

In this study, the MaxEnt model was used to determine the suitable habitat distribution areas of *L. virgaurea* and *L. sagitta*, explore the relationship between environmental variables and these two poisonous plant species, and evaluate the influence of various environmental parameters on the distribution of *Ligularia*. Specifically, there were three key objectives: (1) to predict the distribution pattern of potential suitable areas for *L. virgaurea* and *L. sagitta* under the current climate conditions, and to divide them into different suitability classes; (2) to analyze the predicted relationships between the potential distribution areas of *L. virgaurea* and *L. sagitta* and the main environmental factors; and (3) to predict and compare the potential suitable areas and trends of change in *L. virgaurea* and *L. sagitta* under different climatic conditions in the 2050s and 2070s. The overarching aim in carrying out this work was to provide suggestions for the monitoring and control of poisonous plants in natural grasslands, as well as provide a theoretical basis for coping with the risks of species invasions under future climate change and human disturbance.

## 2 Materials and methods

### 2.1 Study area

**Figure 1:**
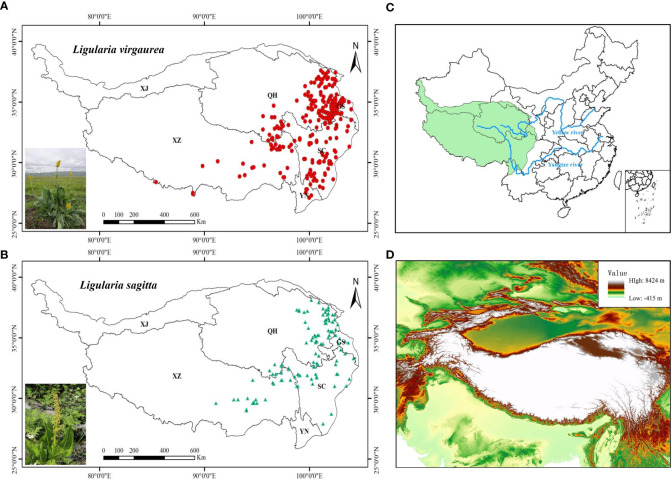
**(A, B)** Sampling locations of two poisonous plants on the QTP: **(A)** *L. virgaurea*; **(B)** *L. sagitta*. **(C)** Map of China showing the location of the QTP study area. **(D)** Digital elevation map of the study area. Province names are abbreviated as follows: XJ, Xinjiang; XZ, Xizang; QH, Qinghai; GS, Gansu; SC, Sichuan; YN, Yunnan.

The Qinghai–Tibet Plateau accounts for about 26.8% of China’s land area, covering the region of 26°00′12″–39°46′50″N and 73°18′52″–104°46′59″E (**Figure 1D**). Topographically, the QTP is high in the northwest and low in the southeast, with an average altitude of over 4000 m, a total area of about 2.5 million square kilometers, and complex and diverse landform types. There are significant regional differences in climate on the QTP, with temperatures showing a gradual decrease from south to northwest, and precipitation a distribution of more precipitation in the southeast and less in the northwest. The QTP is sensitive to climate change and is an ideal place for researching how alpine vegetation systems respond to global changes.

### 2.2 Species occurrence data

The following methods/data sources were used to collect data on the occurrence of *L. virgaurea* and *L. sagitta*: (1) From 2019 to 2021, a field survey was conducted using GPS to record elevation, longitude, and elevation data; (2) the Global Biodiversity Information Facility (www.gbif.org/); (3) the National Specimen Information Infrastructure (http://www.nsii.org.cn/2017/); and (4) the China National Knowledge Infrastructure (http://www.cnki.net/). We collected a total of 465 data on the occurrence of *L. virgaurea* on the QTP, and 128 on the occurrence of *L. sagitta*. These distribution data of *L. virgaurea* and *L. sagitta* on the QTP were then filtered using ArcGIS 10.2 to ensure that each 10×10 km grid cell contained only one record, which ultimately gave us 276 occurrences of *L. virgaurea* and 118 occurrences of *L. sagitta* for subsequent analysis (**Figure 1**).

### 2.3 Environmental variables

We selected 27 environmental variables for the model to predict the probability distributions of *L. virgaurea* and *L. sagitta* on the QTP (**Table 1**), including 19 biological variables, three topographical variables, three soil variables, and two human activity variables. More specifically, to depict the current climatic situation, 19 biological variables from the WorldClim database, version 2.0 (https://www.worldclim.org/), were used with a spatial resolution of 30 arc-seconds, while Geospatial Data Cloud (https://www.gscloud.cn/) provided the three topographical variables, the National Tibetan Plateau Data Center (https://data.tpdc.ac.cn/home) provided the three soil variables, and the Human Footprint Index and actual livestock carrying capacity (https://data.tpdc.ac.cn/home) were the two human activity variables (Duan and Luo, 2021; Liu, 2021).

**Table 1:** Climatic, topographical and soil variables used for modeling climatic niches.

Type	Variable name	Code	Resolution	Year
Climatic	Annual mean temperature	Bio1	1 km	2020
	Mean diurnal range (monthly mean (max temp minus min temp))	Bio2		
	Isothermality (BIO2/BIO7) (×100)	Bio3		
	Temperature seasonality (standard deviation ×100)	Bio4		
	Max temperature of warmest month	Bio5		
	Min temperature of coldest month	Bio6		
	Temperature annual range (BIO5 minus BIO6)	Bio7		
	Mean temperature of wettest quarter	Bio8		
	Mean temperature of driest quarter	Bio9		
	Mean temperature of warmest quarter	Bio10		
	Mean temperature of coldest quarter	Bio11		
	Annual precipitation	Bio12		
	Precipitation of wettest month	Bio13		
	Precipitation of driest month	Bio14		
	Precipitation seasonality (coefficient of variation)	Bio15		
	Precipitation of wettest quarter	Bio16		
	Precipitation of driest quarter	Bio17		
	Precipitation of warmest quarter	Bio18		
	Precipitation of coldest quarter	Bio19		
Topographical	Elevation	Elevation	30 m	2020
	Slope	SLOP		
	Aspect	ASPE		
Soil	Soil total nitrogen	S_TN	250 m	2015-2024
	Soil organic carbon	S_SOC		
	pH	S_PH		
Human activity	Human Footprint Index	HFP	1 km	2017
	Livestock carrying capacity	LCC		2019

Future climatic variables were projected for two future periods (the 2050s and 2070s) using the BCC-CSM2-MR global climate model (GCM) under the conditions of four Shared Socioeconomic Pathways (SSPs) in CMIP6 (phase 6 of the Coupled Model Intercomparison Project): SSP126 (low GHG emissions: CO_2_ emissions cut to net zero around 2075); SSP245 (intermediate GHG emissions: CO_2_ emissions around current levels until 2050, then falling but not reaching net zero by 2100); SSP370 (high GHG emissions: CO_2_ emissions double by 2100); and SSP585 (very high GHG emissions: CO_2_ emissions triple by 2075). BCC-CSM2-MR was chosen because, based on nine widely used GCMs (BCC-CSM2-MR, CNRM-CM6-1, CNRM-ESM2-1, CanESM5, GFDL-ESM4, IPSL-CM6A-LR, MIROC-ES2L, MIROC6, and MRI-ESM2-0), researchers found that, when compared to its previous-generation (CMIP5) version, BCC-CSM2-MR greatly improved the simulation of temperature and precipitation changes in China, and more so than the other eight tested models (Xin et al., 2018).

Many variables exhibit spatial collinearity, which can lead a model to suffer from overfitting, which will ultimately impact the prediction outcomes (Hu and Liu, 2014). In order to filter the environmental variables, we applied Pearson correlation analysis to the 27 environmental variables. We then retained those variables with |r|<0.8, according to the Pearson correlation principle (Fang et al., 2021). This meant that, ultimately, eight climatic variables, three topographical variables, three soil variables, and two human activity variables were selected to participate in the model (**Table 2**). Finally, we used the ArcGIS resampling tool to resample the resolution of the 16 selected environmental variable layers to 1 km for model analysis.

**Table 2:** The relative contributions (%) of variables to the *L. virgaurea* and *L. sagitta* results in the MaxEnt model.

*L. virgaurea*			*L. sagitta*		
Variable	Contribution rate (%)	Cumulative contribution rate (%)	Variable	Contribution rate (%)	Cumulative contribution rate (%)
HFP	52.8	52.8	HFP	42.2	42.2
S_TN	18.9	71.7	Bio13	25	67.2
Elevation	11	82.7	Bio15	7.3	74.5
Bio15	5.1	87.8	Elevation	4.4	78.9
LCC	3.7	91.5	S_TN	4.2	83.1
Bio6	2.5	94	SLOP	2.7	85.8
Bio3	1.9	95.9	ASPE	2.7	88.5
SLOP	1.3	97.2	LCC	2.5	91
S_PH	0.8	98	S_SOC	2.2	93.2
Bio2	0.8	98.8	Bio5	2.1	95.3
ASPE	0.5	99.3	Bio3	2	97.3
S_SOC	0.4	99.7	S_PH	2	99.3
Bio14	0.3	100	Bio2	0.7	100

### 2.4 Model analysis

To predict the possible habitat distributions of *L. virgaurea* and *L. sagitta* on the QTP under climate change, the MaxEnt model, which is a model based on the theory of maximum entropy, was selected. More specifically, we used version 3.4.4 of MaxEnt to model the distribution points of *L. virgaurea* and *L. sagitta* on the QTP, as well as 13 environmental variables, and randomly selected 75% of the species distribution points as the training data and 25% as the test data. We then repeated the procedure 15 times using the cross-validation approach, wherein the maximum number of iterations was 1000, the output format was logistic, and an ASCII file was the final output (Guo et al., 2016).

### 2.5 Model evaluation and validation

The area under the ROC (receiver operator characteristic) curve value was used to measure model performance, which is a threshold-independent approach used to identify presence from absence in species occurrence (Katz and Zellmer, 2018). A model’s accuracy can be judged as excellent if the area under curve (AUC) value is between 0.9 and 1, good if AUC is between 0.8 and 0.9, fair if AUC is between 0.7 and 0.8, poor if AUC is between 0.6 and 0.7, and failed if AUC is between 0.5 and 0.6 (Phillips et al., 2006). The true skill statistic (TSS) has a value ranging from −1 to 1. The higher the TSS value, the greater the consistency between observed and predicted values, and the better the model effect; the lower the TSS value, the worse the consistency and the worse the model prediction impact (Fang et al., 2021). On this basis, the model’s performance was assessed using a combination of AUC and TSS.

### 2.6 Assessment of the importance of environmental variables and classification of suitable habitats

The key ecological constraints restricting the ranges of *L. virgaurea* and *L. sagitta* on the QTP were identified by calculating the contribution of each environmental variable to the expected results in MaxEnt using the knife cut technique (Li et al., 2021). The ASCII files were imported into ArcGIS10.2 and transformed into raster data before being classified into four groups using the spatial analysis tool “reclassify”—namely, unsuitable habitat, low habitat suitability, medium habitat suitability, and high habitat suitability. The distributions and extents of appropriate habitats for *L. virgaurea* and *L. sagitta* on the QTP were determined.

### 2.7 Environmental variable regression analysis and distribution site habitat suitable index

To clarify the relationship between the predicted suitable habitat index and environmental variables of *L. virgaurea* and *L. sagitta*, this study used regression analysis. ArcGIS was used to extract the environmental data and the suitable habitat index of the distribution points. SPSS23 was used for regression analysis of key environmental variables whose cumulative contribution rate to the extracted data and the model was greater than 90%.

## 3 Results

### 3.1 MaxEnt and its accuracy

The average AUC and TSS values of model runs for *L. virgaurea* and *L. sagitta* were evaluated in the Maxent model (**Table 3**). The results show that the prediction of the potential distribution areas of *L. virgaurea* and *L. sagitta* under current and future climatic conditions were highly accurate and performed extremely well. Under current environmental conditions, the mean AUC and TSS values of the *L. virgaurea* and *L. sagitta* model runs were (0.934, 0.922) and (0.943, 0.909), respectively. Furthermore, the average AUC and TSS values of the *L. virgaurea* and *L. sagitta* model runs were greater than 0.88, indicating that they were more accurate and performed better

**Table 3:** The average AUC and TSS values of the model runs.

Time	Emission scenarios	*L. virgaurea*		*L. sagitta*	
		Training AUC	Test AUC	Training AUC	Test AUC
Current		0.934	0.922	0.943	0.909
2050s	SSP126	0.904	0.888	0.920	0.897
	SSP245	0.904	0.892	0.922	0.901
	SSP370	0.906	0.890	0.924	0.901
	SSP585	0.905	0.891	0.921	0.898
2070s	SSP126	0.902	0.887	0.924	0.902
	SSP245	0.905	0.890	0.923	0.901
	SSP370	0.907	0.893	0.924	0.900
	SSP585	0.905	0.889	0.922	0.900

### 3.2 Key environmental variables

We obtained the contribution rate of each variable in the MaxEnt model and analyzed the environmental variables that had a greater impact on the prediction result. The cumulative contribution rate of the four types of variables was calculated (**Table 2**). Climatic variables accounted for 10.6% and 37.1% of the result for *L. virgaurea* and *L. sagitta*, respectively. Meanwhile, topographical variables accounted for 12.8% and 9.8%, soil variables accounted for 20.1% and 8.4%, and human activity variables accounted for 56.5% and 44.7%, respectively. The proportions for the human activity variables were larger, demonstrating that they had a greater impact on the model prediction results. In summary, human activity variables had the greatest impact on the potential distribution of *L. virgaurea* and *L. sagitta*, followed by climatic variables, while topography variables had the least influence.

### 3.3 Regression analysis of environmental variables

The key factors, which had a cumulative contribution rate of more than 90% to the MaxEnt model, were used for regression analysis along with the suitable habitat index of distribution points (**Table 4**). HFP and elevation had substantial (*P*<0.01) influences on the distribution of both *L. virgaurea* and *L. sagitta*, whilst S_TN had no effect. Bio15 had significant (*P*<0.01) impacts on the distribution of *L. virgaurea* and *L. sagitta*, respectively, whereas Bio13 had significant (*P*<0.01) effects on the distribution of *L. sagitta*, and Bio15, SLOP and ASPE had no significant influence on the distribution of *L. sagitta*. This indicates that human activity is the primary factor influencing the spread of *L. virgaurea* and *L. sagitta* on the QTP and that the distribution area and range of these two poisonous plants has expanded as the HFP intensity has increased.

**Table 4:** Regression analysis of important environmental factors and distribution site habitat suitability index.

L. virgaurea				L. sagitta			
Variable	Slope	R^2^	P	Variable	Slope	R^2^	P
HFP	0.011	0.09	<0.01	HFP	0.015	0.418	<0.01
S_TN	0.009	0.001	0.658	Bio13	−0.004	0.096	<0.01
Bio15	−0.006	0.038	<0.01	Bio15	0.004	0.013	0.224
Elevation	−0.0002	0.144	<0.01	Elevation	−0.001	0.309	<0.01
–	–	–	–	S_TN	−0.023	0.07	0.379
–	–	–	–	SLOP	-0.0008	0.004	0.049
–	–	–	–	ASPE	-0.0001	0.001	0.886

### 3.4 Potential distribution of *L. virgaurea* and *L. sagitta* in the current climate

**Figure 2:**
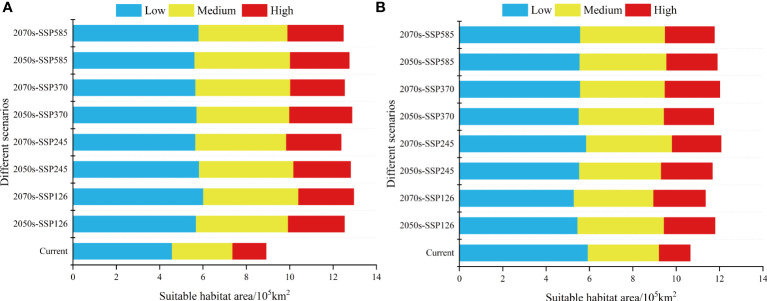
Change in the range of suitable habitat area for **(A)** *L. virgaurea* and **(B)** *L. sagitta* under current and future climate scenarios.

**Figure 3:**
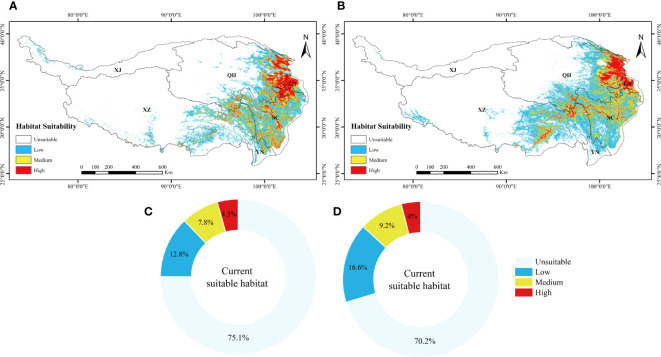
The suitable distribution area of **(A)** *L. virgaurea* and **(B)** *L. sagitta* predicted by MaxEnt on the QTP under current climatic conditions. Panels **(C, D)** show the proportions of the different grades of current suitable habitat for *L. virgaurea* and *L. sagitta* on the QTP, respectively.

For the present day, the results show that the suitable invasive areas for *L. virgaurea* and *L. sagitta* are mainly distributed in the eastern and southwestern part of the QTP, southwestern Gansu, eastern Qinghai, western Sichuan, southeastern Tibet, and northwestern Yunnan, with a total area of 8.92×10^5^km^2^ and 10.65×10^5^km^2^ (**Figures 2A, B**), respectively. Under the current climatic scenario, the potential distribution areas of *L. virgaurea* and *L. sagitta* account for 12.8%, 7.8%, 4.3%, 16.6%, 9.2%, and 4% of the total area of the QTP, respectively, and are mostly concentrated in Qinghai, Gansu, Sichuan, and Tibet (**Figure 3**).

### 3.5 Impacts of future climate scenarios on the potential distribution of *L. virgaurea*

**Figure 4:**
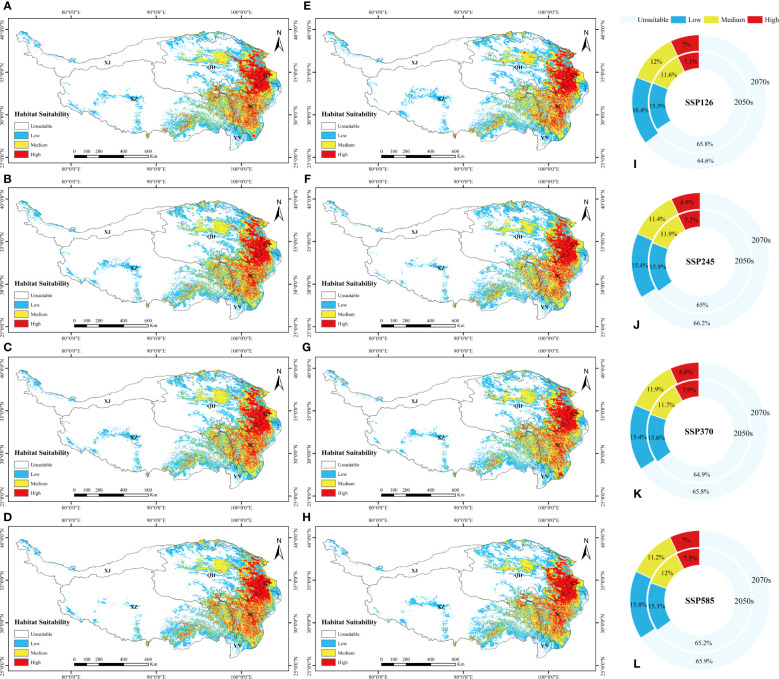
The suitable distribution area of *L. virgaurea* predicted by MaxEnt on the QTP in the **(A-D)** 2050s and **(E-H)** 2070s, under **(A, E)** SSP126, **(B, F)** SSP245, **(C, G)** SSP370, and **(d, h)** SSP585. Panels **(I-L)** show the area ratios under the four scenarios, respectively, for the 2050s and 2070s separately.

The total potential distribution area of *L. virgaurea* was projected to increase substantially over time under the four future emission scenarios. Specifically, the area accounted for 34.2%, 35%, 35.1% and 34.8% of the total area of the QTP in the 2050s under SSP126, SSP245, SSP370 and SSP585, respectively; and 32.2%, 31.8%, 32% and 32.5% in the 2070s (**Figure 4**). The area under the SSP126 scenario increased the most from the 2050s to the 2070s, whereas it decreased under the other three scenarios. For the 2070s under SSP126, the overall area expanded the largest when compared to the existing distribution area, reaching 12.96×10^5^km^2^ (**Figure 2A**). From the 2050s to the 2070s, the area of high habitat suitability increased and then decreased, with the largest extent in the 2050s under SSP370, at 2.89×10^5^km^2^ (**Figure 2A**), while the area of medium habitat suitability increased and then decreased. In comparison to the existing distribution area, the prospective distribution region of *L. virgaurea* on the QTP was projected to expand owing to the effects of future climate change, with the amount of appropriate habitat peaking in the 2050s, mostly concentrated on the QTP. These locations are expected to be more favorable for the growth and reproduction of *L. virgaurea* in the face of climate change.

### 3.6 Impacts of future climate scenarios on the potential distribution of *L. sagitta*

**Figure 5:**
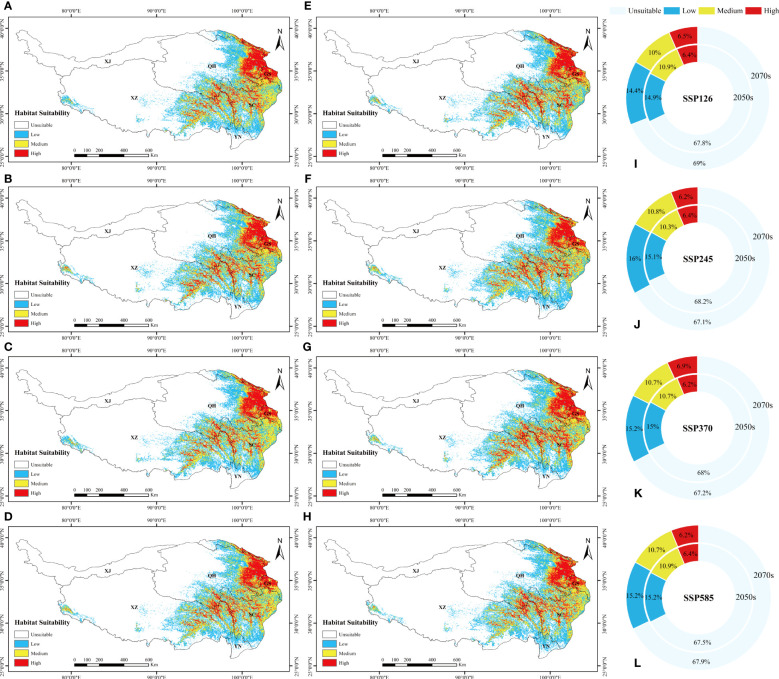
The suitable distribution area of *L. sagitta* predicted by MaxEnt on the QTP in the **(A–D)** 2050s and **(E–H)** 2070s, under **(A, E)** SSP126, **(B, F)** SSP245, **(C, G)** SSP370, and **(D, H)** SSP585. **(I–L)** show the area ratios under the four scenarios, respectively, for the 2050s and 2070s separately.

The total potential distribution area of *L. sagitta* was projected to grow greatly over time under the four future emission scenarios. Specifically, the area accounted for 32.2%, 31.8%, 32% and 32.5% of the total area of the QTP in the 2050s under SSP126, SSP245, SSP370 and SSP585, respectively; and 31%, 32.9%, 32.8% and 32.1% in the 2070s (**Figure 5**). The area under SSP245 increased the most from the 2050s to the 2070s, followed by those under SSP370, SSP126 and SSP585, which all produced a decrease in the distribution area. For the 2070s under SSP370, the total area expanded the greatest when compared to the existing distribution area, reaching 12.01×10^5^km^2^ (**Figure 2B**). From the 2050s to the 2070s, the area of high habitat suitability increased gradually, with a maximum area of 2.53×10^5^km^2^ for the 2070s under SSP370, and the amount of medium habitat suitability decreased and subsequently increased under SSP245, SSP126 and SSP370 (**Figure 2B**). Under the effects of future climate change, the prospective distribution region of *L. sagitta* on the QTP is expected to expand in comparison to the existing distribution area. Under future climatic conditions, *L. sagitta* will likely be primarily distributed in the eastern and southeastern parts of the QTP, and the distribution areas of high habitat suitability will primarily be in southeastern Qinghai, indicating that these locations are more suitable for the growth of *L. sagitta*, and their climate is the primary reason for their expected expansion.

## 4 Discussion

As an important part of the terrestrial ecosystem, grassland plays an important role in regulating climate, nourishing water, and maintaining species diversity (Dong et al., 2022). However, when grassland is overused, poisonous plants that are not easily foraged by herbivores and can adapt to drastic climate change can invade. They spread rapidly in grassland, outcompeting other grass and sedge plants for light, water, nutrients, and other resources to gradually become the dominant species in the community. This has led to a decline in grassland production and community diversity and the availability of soil nutrients in grassland ecosystems, which seriously affects the sustainability of cycles in grassland biomes (Lu et al., 2012; Ren et al., 2013). The QTP has the most different types of grasslands in China, as well as the largest area (Peng et al., 2020). Poisonous plants will continue to spread on the QTP as a result of climate change if effective management and interventions are not implemented. Our research provides an important reference for dealing with the expansion of poisonous plants on the QTP under climate change.

### 4.1 The main factors currently affecting the distribution of *L. virgaurea* and *L. sagitta* on the QTP

#### 4.1.1 Climatic variables

Precipitation and temperature are the main factors affecting plant growth and reproduction (Zhang et al., 2021; Yang et al., 2022a). Moreover, global climate change has resulted in more extreme variations in precipitation. Due to the complex terrain and elevation of the QTP, there are large spatial differences in precipitation and temperature (Körner et al., 2016). Plants growing in high-elevation areas of the QTP need to cope with extreme climatic events such as drought and low temperatures, which cause the plant seed germination rate to decrease, biomass to decrease, and survival strategies to change. Therefore, plants that have adapted to extreme climate change are more likely to become dominant species that reproduce and spread rapidly in grasslands (Bita and Gerats, 2013; Dürr et al., 2017). According to the findings of this study, the predicted contributions of precipitation to the distribution of *L. virgaurea* and *L. sagitta* were 5.4% and 32.3%, respectively, while those of temperature were 5.2% and 4.8%. Bio15 was the main climatic variable found to influence the distribution of *L. virgaurea* and *L. sagitta* on the QTP, accounting for 5.1% and 7.3% of the model predictions, respectively, and the habitat suitability index of *L. virgaurea* showed a significant decreasing trend with an increase in Bio15, whereas the habitat suitability index of *L. sagitta* was not significantly related to Bio15. The distribution of *L. sagitta* was mainly affected by Bio13, and the contribution rate was 25% in the model prediction. Both plant species were mainly distributed in the range of 80–100 mm for Bio15, with the most suitable habitat being near to 90 mm; and for Bio3, *L. sagitta* was mainly distributed within 75–200 mm, with the most suitable habitat being near to 100 mm. Seasonally, precipitation on the QTP is high in the east and low in the west (Li et al., 2022), and *L. virgaurea* and *L. sagitta* are mainly distributed in the eastern region where there is low precipitation. The two plants are raceme plants with dense flowers, for which studies have shown that excessive precipitation during the flowering period may lead to inflorescence shedding, which is not conducive to the results (Nakata et al., 2022). Precipitation on the QTP is mainly concentrated in summer; and if precipitation is too strong, it will affect the results of the two plants. Therefore, areas with low precipitation are more conducive to successful reproduction in these two plant species.

#### 4.1.2 Topographical variables

The results of this study showed that the contribution rates of topographical variables to the predicted distributions of *L. virgaurea* and *L. sagitta* were 12.8% and 9.8%, respectively. Elevation was found to be an important factor affecting the distribution of *L. virgaurea* and *L. sagitta*, contributing 11% and 4.4% to the model prediction, respectively. The habitat suitability index of the two plants decreased significantly as elevation increased, being mainly distributed between 1000 and 4000 m. The QTP is a high-elevation area with considerable day and night temperature differences, and plant growth is sensitive to temperature changes (Dai et al., 2019). Plants occupy a dominant position in the community through reproduction and dispersal and maximizing energy allocation to nutritional reproduction ensures that plants generate more seeds (He et al., 2017). For perennial herbs, growth in high-elevation areas requires more energy to overcome low temperatures to ensure survival (Jin et al., 2022). High elevation causes a change in allocation strategy, directing more energy to growth rather than vegetative reproduction, which in the present case would have a negative impact on the reproduction and dispersal of these two plants (Lubbe et al., 2021).

#### 4.1.3 Soil variables

Soil is a crucial substrate for plant growth and reproduction, and among the soil nutrients that are essential for these processes, nitrogen (N) is key (Kulmatiski et al., 2008). In this study, the contribution rates of soil variables to the predicted distributions of *L. virgaurea* and *L. sagitta* were 20.1% and 8.4%, respectively, of which N accounted for 18.9% and 4.2%, respectively. Nitrogen is an important component of chlorophyll in plant leaves and plays a crucial role in plant photosynthesis. Compared with other herbaceous plants on the QTP, *L. virgaurea* and *L. sagitta* are tall and possess larger leaves, both of which are characteristics that are more conducive to photosynthesis and have a greater demand for N during growth and reproduction (Song et al., 2022). The soil N content of *L. virgaurea* and *L. sagitta* increased with density in the distribution area, with higher N content in the southeast and northwest of the QTP, consistent with the predicted distribution areas of *L. virgaurea* and *L. sagitta* in this study (Liu et al., 2022a; Wang et al., 2022). Consequently, high N soils provide important elements for the growth of *L. virgaurea* and *L. sagitta*, as well as promote photosynthesis, providing extremely favorable basic conditions for reproduction and dispersal.

#### 4.1.4 Human activity

Due to rapid socioeconomic development, the intensity and range of human activities on the QTP have gradually increased, and the impact on the ecosystem has gradually strengthened (Zhu et al., 2022). When predicting the distribution area and range of poisonous plants on a large scale, it is necessary to add human activity intensity data for modeling and prediction, and it has been found that doing so improves the accuracy of prediction results (Yang et al., 2022a). In this study, human activity contributed 52.8% and 42.2% to *L. virgaurea* and *L. sagitta*, respectively, which increased significantly with an increase in human activity intensity. Regression analysis showed that there was a significant positive correlation between the intensity of human activities *L. virgaurea* and *L. sagitta*. The contribution rate in the model prediction was the largest, which can better explain that the intensity of human activities is the driving factor for the large-scale expansion of plants in recent years (Xu et al., 2019). Based on the above results, it can be inferred that a series of human activities such as land use, infrastructure construction, population density, roads and railways will have a positive impact on the growth, habitat and reproduction of *L. virgaurea* and *L. sagitta* (Yang et al., 2022a).

### 4.2 Changes in distribution areas under future climate change

In this study, the climate factors and topographical factors of four emission scenarios (SSP126, SSP245, SSP370, and SSP585) under future climate change were adopted, and the MaxEnt model was used to simulate and predict the potential distribution areas and distribution ranges of *L. virgaurea* and *L. sagitta* on the QTP against the background of future climate change from the 2050s to 2070s. The results showed that, under the four emission scenarios, the distribution areas and distribution ranges of different habitats of *L. virgaurea* and *L. sagitta* increased and decreased with the change in future climate. This demonstrates that future climate change will have an important impact on the potential distribution range and suitable habitat of these two plant species. Among all the climatic factors, Bio15 and Bio13 were the most important, indicating that the growth and distribution of *L. virgaurea* and *L. sagitta* are strongly related with precipitation and temperature. Against the future climate background, with the increase in emissions intensity, the precipitation and temperature of the QTP will increase, and the change in temperature will be obviously different at different elevations. The overall finding was that the temperature increase in the high-elevation areas of the northern QTP will be greater than that in the low-elevation areas of the southeastern QTP; and meanwhile, the most significant increase in precipitation is expected in the northwest region (Luo et al., 2022). Plant growth is determined by water and temperature, and when drought and annual accumulated temperature change, plant growth will be severely limited (Ganjurjav et al., 2016). The growth and distribution of plants has an important relationship with soil nutrients. The soil TN content of high-density *L. virgaurea* and *L. sagitta* communities is higher. In the future, with the increase in temperature, the soil biological activities related to soil N transformation will be promoted, which will accelerate the soil N cycle, increase the content of soil available N, and further promote the growth and distribution of *L. virgaurea* and *L. sagitta* (Mei and Ma, 2021). The MaxEnt simulation predicted the geographical distribution of *L. virgaurea* and *L. sagitta* on the QTP in the context of future climate change, and this information can be used to monitor and provide advanced warnings regarding the occurrence of these poisonous plants, as well as serve as a reference for the formulation of prevention and control measures in key areas.

In summary, this study predicted the current and future distribution of *L. virgaurea* and *L. sagitta* through the MaxEnt model. Although there are a few studies that have reported the distribution of poisonous plants and predicted the potential distribution of poisonous plants at local scales in some regions under climate change (Huang et al., 2020), there is, however, a lack of reports on the widespread distribution of these two poisonous plants on the QTP, especially considering the joint effects of climate change and human disturbance. Our study provides a basis for understanding environmental factors and the reasons for the widespread distribution of two poisonous plants, as well as serving as a reference for managing the spread of poisonous plants in the future. However, we only considered the role of abiotic factors in predicting the spread of poisonous plants. It is also very important to study the role of biological factors, such as plant intraspecific and interspecific competition, in predicting the spread of poisonous plants in the future.

## 5 Management

Based on the findings of this study, it is suggested that current management should focus on monitoring areas with habitats of high and medium suitability, and then implement control measures in a timely manner. Under future climate change, monitoring work should be carried out on the suitable potential expansion areas of *L. virgaurea* and *L. sagitta*. At the same time, human disturbance should be reduced to inhibit the growth and spread of *L. virgaurea* and *L. sagitta* in areas of potential occurrence. The QTP is an important gene bank of species in China, and the prevention and control of poisonous plants plays a key role in maintaining species diversity. This study used SDM simulations to predict the geographical distributions of *L. virgaurea* and *L. sagitta* on the QTP against the background of future climate change. Monitoring and early warning of plant occurrence areas can serve as a reference for the early formulation of prevention and control work, as well as meet the needs of large-scale and small-area monitoring work, which has certain theoretical and practical significance.

## 6 Conclusion

Based on the MaxEnt model, this study selected five climatic, three topographical, three soil, and two human activity variables to analyze the predicted geographical distributions and occurrences areas of *L. virgaurea* and *L. sagitta*. The results showed that human activity and rainfall are the main factors limiting the current distribution range of these two species. Under the current climate, the potential distribution areas of *L. virgaurea* and *L. sagitta* on the QTP were found to be 8.92×10^5^km^2^ and 10.65×10^5^km^2^, respectively, accounting for approximately 24.9% and 29.8% of the total QTP area, mainly concentrated in southern Gansu, eastern Qinghai, northwest Sichuan, northwest Yunnan, central Tibet, and southwest Xinjiang. Both human footprint index and elevation were significantly positively correlated with the distribution of *L. virgaurea* and *L. sagitta*, while rainfall was significantly negatively correlated. Under the four future climate scenarios, compared with their current distribution areas, the distribution areas of *L. virgaurea* and *L. sagitta* were projected to increase in the 2050s to 2070s, particularly the suitable distribution area of *L. virgaurea*. The largest area, up to 12.96×10^5^km^2^, and the total area of *L. sagitta*, increased the most in the 2070s under SSP370, up to 12.01×10^5^km^2^.

## Data availability statement

The original contributions presented in the study are included in the article/supplementary material. Further inquiries can be directed to the corresponding author.

## Author contributions

RD and L-MH: conception and design of the research RD, X-CC, Z-ST X-CZ and BC: acquisition of data RH and BC: analysis and interpretation of data. G-HY and DB: statistical analysis RD and L-MH: drafting the manuscript. All authors contributed to the article and approved the submitted version.
